# The Thermostable Direct Hemolysin from *Grimontia hollisae* Causes Acute Hepatotoxicity *In Vitro* and *In Vivo*

**DOI:** 10.1371/journal.pone.0056226

**Published:** 2013-02-21

**Authors:** Yan-Ren Lin, Yao-Li Chen, Keh-Bin Wang, Yi-Fang Wu, Yu-Kuo Wang, Sheng-Cih Huang, Tzu-An Liu, Manoswini Nayak, Bak-Sau Yip, Tung-Kung Wu

**Affiliations:** 1 Department of Biological Science and Technology, National Chiao Tung University, Hsin-Chu, Taiwan, Republic of China; 2 Department of Emergency Medicine, Changhua Christian Hospital, Changhua, Taiwan, Republic of China; 3 Transplant Medicine and Surgery Research Centre, Changhua Christian Hospital, Changhua, Taiwan, Republic of China; 4 Department of Nuclear Medicine, Kuang Tien General Hospital, Taichung, Taiwan, Republic of China; 5 Institute of Cellular and System Medicine, National Health Research Institutes, Zhunan, Miaoli County, Taiwan, Republic of China; 6 Department of Neurology, National Taiwan University Hospital Hsin Chu Branch, Hsin-Chu, Taiwan, Republic of China; 7 School of Medicine, Chung Shan Medical University, Taichung, Taiwan, Republic of China; University of Leicester, United Kingdom

## Abstract

**Background:**

*G. hollisae* thermostable direct hemolysin (Gh-TDH) is produced by most strains of *G. hollisae.* This toxin has been reported to be absorbed in the intestines in humans. Secondary liver injury might be caused by venous return of the toxin through the portal system. We aimed to firstly analyze the *in vitro* and *in vivo* hepatotoxicity of Gh-TDH.

**Methods:**

Liver cells (primary human non-cancer cell and FL83B mouse cells) were treated and mice (BALB/c) were fed with this toxin to investigate its hepatotoxicity. Morphological examination and cytotoxicity assays using liver cells were also performed. Fluorescein isothiocyanate-conjugated toxin was used to analyze the localization of this protein in liver cells. Mice were subjected to liver function measurements and liver biopsies following toxin treatment and wild-type bacterial infection. PET (positron emission tomography)/CT (computed tomography) images were taken to assess liver metabolism during acute injury and recovery.

**Results:**

The effect of hepatotoxicity was dose and time dependent. Cellular localization showed that the toxin was initially located around the cellular margins and subsequently entered the nucleus. Liver function measurements and liver biopsies of the mice following treatment with toxin or infection with wild-type *Grimontia hollisae* showed elevated levels of transaminases and damage to the periportal area, respectively. The PET/CT images revealed that the reconstruction of the liver continued for at least one week after exposure to a single dose of the toxin or bacterial infection.

**Conclusions:**

The hepatotoxicity of Gh-TDH was firstly demonstrated. The damage was located in the periportal area of the liver, and the liver became functionally insufficient.

## Introduction

Diseases caused by different *Vibrio* species have been observed in large populations throughout the world, particularly in Asia, the United States, and Africa [1]–[3]. *V. cholera* and *V. parahaemolyticus* are the major etiological agents of vibriosis, which is associated with the ingestion of raw, undercooked, or contaminated seafood [1], [2]. *Grimontia hollisae* (previously named *V. hollisae)* has been frequently reported to cause diseases in humans, including severe gastroenteritis, hypovolemia, and septicemia following the consumption of shellfish or oysters [4]–[6]. However, the mechanism of morbidity due to *G. hollisae* has not been thoroughly characterized. Although several specific laboratory examinations have detected *G. hollisae*, its incidence is likely highly underestimated because detection techniques are infrequently used throughout the world [7], [8].

The toxin thermostable direct hemolysin (TDH) is composed of 165 amino acid residues and exhibits biological activities that include hemolytic activity, cytotoxicity, and enterotoxicity [9]. The toxic effects of TDH have been identified in a variety of *Vibrio* species, including *V. cholera* non-O1, *V. parahaemolyticus*, *V. mimicus*, *V. alginolyticus*, and *G. hollisae* [10]–[14]. In addition, TDH production has been observed in bacteria with similar *tdh* gene sequences and pathogenicities [15], [16]. The *tdh* gene is present in all strains of *G. hollisae* but not in all *Vibrio* species [10]. The physical characteristics of *G. hollisae* TDH (Gh-TDH) are not well known. Some studies have reported that Gh-TDH is detoxified by aggregation into fibrils after heating at 60–70°C; the protein can be reversibly refolded into the toxic native form by rapid cooling after unfolding at higher temperatures [17]. The hemolytic activity of Gh-TDH is suppressed by the addition of Congo red [18]. In addition, the lipophilic effect of Gh-TDH has not been clearly characterized. The toxic effects of TDH have been localized mainly in the intestinal portion of the gastrointestinal tract [19]–[21]. However, a relationship between the TDH and the liver has not been reported or analyzed. In addition to being absorbed by the intestine, TDH may also cause secondary injury to the liver via effects on the venous return of the portal system. In this study, we analyzed the hepatotoxicity of TDH *in vivo* and *in vitro* to provide insights into the acute injury and recovery stages of THD-induced hepatotoxicity in living animals.

## Materials and Methods

### Bacterial Strains and Materials

*G. hollisae* strain ATCC 33564 was obtained from the Culture Collection and Research Center (Hsin-Chu, Taiwan). Phenyl Sepharose 6 Fast Flow and protein molecular weight standards were purchased from GE Healthcare (Piscataway, NJ). The protein assay kit was obtained from Bio-Rad (Hercules, CA). Protein purification chemicals were obtained from Calbiochem (La Jolla, CA).

### Molecular Cloning, Protein Expression and Purification, and Characterization of *G. hollisae* Recombinant Thermostable Direct Hemolysin (Gh-rTDH)

Molecular cloning, protein expression, and purification of Gh-rTDH were performed according to previous publications [20], [21]. The effect of endotoxin was excluded before the start of the experiment. For the purpose of this study, endotoxin contamination was excluded during protein preparation by anion-exchange chromatography using diethylaminoethane (DEAE) chromatographic matrices [22], [23]. The protein identities of the SDS-PAGE bands corresponding to Gh-rTDH were confirmed by MALDI-TOF/TOF spectrometry.

### Cytoviability and Morphological Examination of Gh-rTDH-treated Human Liver Cells and FL83B Cells

FL83B (BCRC 60325) and primary human non-cancer cells (which were kindly provided by the liver transplantation center of a medical center in central Taiwan; IRB number: 120305) were cultured for use in these studies. Following surface attachment, the cells were treated with Gh-rTDH at a concentration of 1 µg/ml for 24 hr at 37°C; the treatment dose was determined by using the initial results from the IC_50_ determination (1 µg/ml, as obtained from the MTT assay). Cellular morphology in the experimental group was observed microscopically at 4 time points (before and after exposure to Gh-rTDH for 8, 16, and 24 hr). Cells treated with PBS (mixed with culture medium) were used as the control group and were observed at the same time points as the experimental group. The cytoviability of human liver cells and FL83B cells was measured by MTT assay at 4 treatment durations (12, 16, 24, and 48 hr). In the MTT assay, cells were treated with PBS as a control and with Gh-rTDH at different concentrations (10 to 10^−8 ^µg/ml mixed with culture medium and administered in a total volume of 250 µl). All experiments were independently performed five times.

### Localization of the Gh-rTDH Protein in FL83B Cells

To investigate the localization of Gh-rTDH after its entry into FL83B cells, Gh-rTDH was conjugated with fluorescein isothiocyanate (FITC) to produce Gh-rTDH-FITC, and reactions were performed using the FluoReporter FITC Protein Labeling Kit (Molecular Probes) according to the manufacturer’s protocol. Two batches of cells (plated at 1×10^4^ cells/well) were independently treated with 10 µg/ml Gh-rTDH-FITC for 20 and 40 min, washed 3 times with PBS, and stained with propidium iodide (PI) for 5 min in the dark. Images were acquired by confocal microscopy (wavelength: λ_ex_ = 488 and λ_em_ = 650 nm).

### *In vivo* Hepatotoxicity of Gh-rTDH in BALB/c Mice

A total of 114 six-week-old female mice were obtained from the National Laboratory Animal Center of Taiwan for the analysis of *in vivo* hepatotoxicity. All mice were fed normal diets. This study was carried out in strict accordance with the recommendations of the Guide for the Care and Use of Laboratory Animals of the National Institutes of Health. The protocol was approved by the Committee on the Ethics of Animal Experiments of National Chiao Tung University (Permit Number: 01001008). All surgery was performed under sodium pentobarbital anesthesia, and all efforts were made to minimize suffering.

### Withdrawal of Blood for Liver Function Analysis (n = 25)

A total of 25 mice were assigned to one of 5 groups (n = 5 for each group). One group served as a control group and was administered PBS; the other 4 groups were administered different doses of Gh-rTDH (0.1, 1, 10, and 100 µg) in a single treatment. The dosage that might initiate organ injury in animals has not been reported (information on natural infection in humans is also lacking). Therefore, the treatment dosages were carefully determined and modified according to the initial results of the IC_50_ determination (1 µg/ml, as determined by the MTT assay described above). All mice were treated with the same volume (200 µl) and the same treatment time (10∶00 a.m.) via gastric tubes without volume loss (i.e., vomiting). A total of 100 µl of whole blood was withdrawn from the orbital vascular plexus of each mouse through a capillary tube with no analgesics. Samples were taken at 8 time points: before treatment with PBS or Gh-rTDH and 4, 8, 16, 32, 64, 128, and 256 hr after treatment with PBS or Gh-rTDH. The blood samples were analyzed for the continuation of liver function as assessed by glutamic-oxaloacetic transaminase (GOT), glutamic-pyruvic transaminase (GPT), total/direct/indirect bilirubin, albumin, and globulin (Reagents Beckman Coulter). One-way ANOVA analysis was used to analyze the significance of differences between each treatment/time point. All analyses were performed with the SPSS statistical package for Windows (Version 15.0, SPSS Inc., Chicago, IL).

### Withdrawal of Blood for Cardiotoxicity and Nephrotoxicity Analyses (n = 20)

A total of 20 mice were assigned to one of 4 groups (n = 5 in each group). One group served as the control group and was treated with PBS. The other 3 groups were treated with Gh-rTDH at doses of 1, 10, and 100 µg in a single administration via a gastric tube. A total of 100 µl of whole blood was withdrawn from each mouse at 5 time points: before treatment with PBS or Gh-rTDH and 4, 16, 64, and 256 hr after treatment with PBS or Gh-rTDH. Nephrotoxicity was assessed by determining the creatinine levels in the blood samples (Creatinine Reagent, Beckman Coulter), and cardiotoxicity was assessed by analyzing the levels of CK-MB (CK-MB Reagent Pack, Beckman Coulter) and troponin I (ADVIA Centaur TnI-Ultra Ready Pack).

### Liver Biopsy (n = 9)

A total of 9 mice were assigned to one of 3 groups which were treated with PBS, 10 µg Gh-rTDH, or 100 µg Gh-rTDH (n = 3 in each group) in a single administration via a gastric tube. The livers of all mice were biopsied after 8 hr of treatment. Samples were prepared from tissue that was harvested at the time of sacrifice and subjected to H&E staining.

### PET/CT Scan (n = 60)

A ^18^F-FDG PET/CT scan was used to detect liver cell glucose metabolism in living animals after exposure to Gh-rTDH to monitor trends in glucose metabolism (GE Medical System). ^18^F-FDG is an analog of glucose that can be used to measure glucose metabolism in organs and cells. A total of 60 mice were assigned to one of 4 dosage groups, and each group (n = 15) was treated with PBS or 1, 10, or 100 µg of Gh-rTDH in a single administration. Within each dosage group, mice were further sub-grouped to receive ^18^F-FDG PET/CT scans over time; examinations were performed at 8, 72, and 168 hr (n = 5 for each time group) after treatment with Gh-rTDH. For this study, 0.07 mCi of ^18^F-FDG was administered to each mouse by tail vein injection. Imaging was performed under appropriate general anesthesia (Isoflurane) one hour after ^18^F-FDG injection. In this study, each mouse did not receive a ^18^F-FDG PET/CT scan at each time point. The recurring general anesthesia might cause hepatotoxicity, which could influence the results of the study. For this analysis, the ^18^F-FDG uptake value was calculated by using a region of interest approach (ROI). The ROIs of liver and muscle (left foot) were determined by a semi-quantitative method using the ratio of liver/muscle ^18^F-FDG uptake.

### Infection Models of *in vivo* Hepatotoxicity of *G. hollisae, Escherichia coli* Expressing Recombinant Gh-tdh (E. coli-TOPO-tdh), and the *E. coli*-TOPO Strain in BALB/c Mice (n = 126)

An animal infection model was established to evaluate the hepatotoxicity of bacterial infection. The *G. hollisae* (wild type), *E. coli-*TOPO*-tdh*, and *E. coli-*TOPO strains were cultured. A total of 75 mice were assigned to one of three major groups (n = 25 for each group) and infected with bacteria via oral administration. Two groups were infected with *G. hollisae* and *E. coli*-TOPO-*tdh* to demonstrate their hepatotoxicity; the third group was infected with *E. coli*-TOPO as a control. For each major group, five subgroups were established (n = 5 for each group) according to treatment dosage (10^7^, 10^8^, 10^9^, 10^10^, and 10^11^ organisms/ml, all with the same volumes). A total of 100 µl of whole blood was withdrawn at 8 different time points: before treatment with bacteria and 4, 8, 16, 32, 64, 128 and 256 hours after bacterial treatment. Blood samples were analyzed for continued liver function (GOT, GPT, total bilirubin, albumin, and globulin). In addition, 6 mice were treated with 10^11^ organisms/ml of *G. hollisae, E. coli*-TOPO-*tdh*, and *E. coli*-TOPO (n = 2 for each group). For these animals, liver biopsies and H&E staining (200X) were performed 8 hr after bacterial treatment. Finally, 54 mice were treated with *G. hollisae, E. coli*-TOPO-*tdh*, and *E. coli*-TOPO (n = 18 for each group) with a single administration. Within each group, mice were sub-grouped for treatment with bacteria at concentrations of 10^7^, 10^9^, and 10^11^ organisms/ml (n = 6 for each group). In each concentration group, mice received a PET/CT scan at 8, 72, and 168 hr (n = 2 for each group) after bacterial treatment.

## Results

### 1. Identification of Gh-rTDH Purified from *G. hollisae*

**Figure 1:**
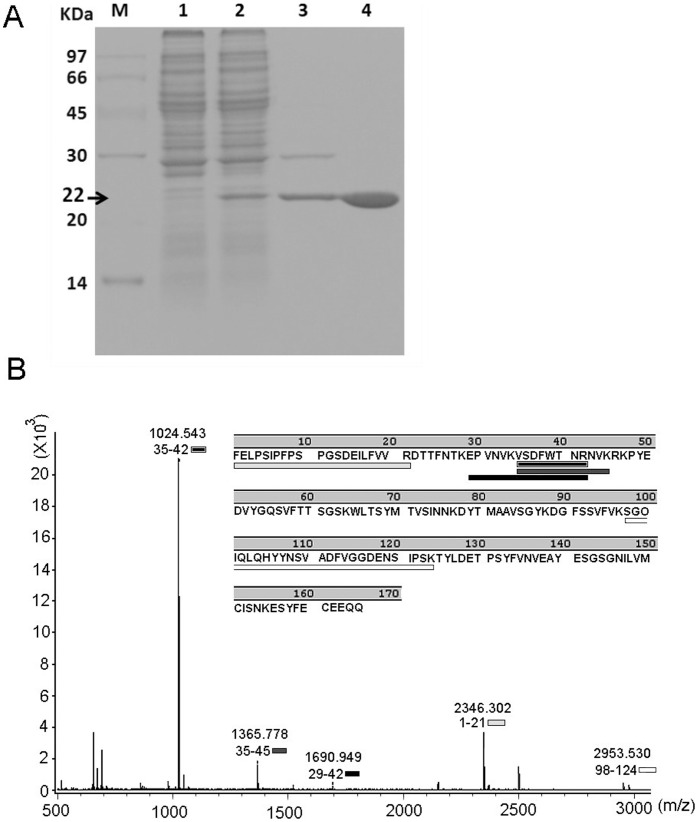
Identification of Gh-rTDH purified from G. hollisae. (A) SDS-PAGE analysis of Gh-rTDH. Marker proteins (M): phosphorylase b (97 kDa), albumin (66 kDa), ovalbumin (45 kDa), carbonic anhydrase (30 kDa), trypsin inhibitor (20 kDa), and α-lactoalbumin (14 kDa); lane 1: cell crude extract of BL21(DE3) pLysS containing the pCR2.1-TOPO plasmid alone; lane 2: crude protein expression in BL21(DE3) pLysS containing pCR2.1-TOPO-*Gh-tdh*; lanes 3 and 4: Phenyl Sepharose 6 Fast Flow purification yielded a homogenous protein with a molecular mass of ∼22 kDa. (B) The tandem mass spectrum of the doubly charged tryptic peptide at *m/z* 1024.543 from the SDS-PAGE of Gh-rTDH revealed a unique hit matching ^35^VSDFWTNR^42^ of the Gh-rTDH peptide sequence.

SDS-PAGE of the homogeneous protein indicated a molecular mass of ∼22 kDa. Moreover, the tandem mass spectrum of the doubly charged tryptic peptide at *m/z* 1024.543 from an SDS-PAGE of Gh-rTDH revealed a unique hit matching ^35^VSDFWTNR^42^ of the Gh-rTDH peptide sequence (by MALDI TOF/TOF spectrum) (Figure 1).

### 2. Hepatotoxicity was Demonstrated by *in vitro* Analyses

#### 2.1 Gh-rTDH causes *in vitro* liver cell damage

**Figure 2:**
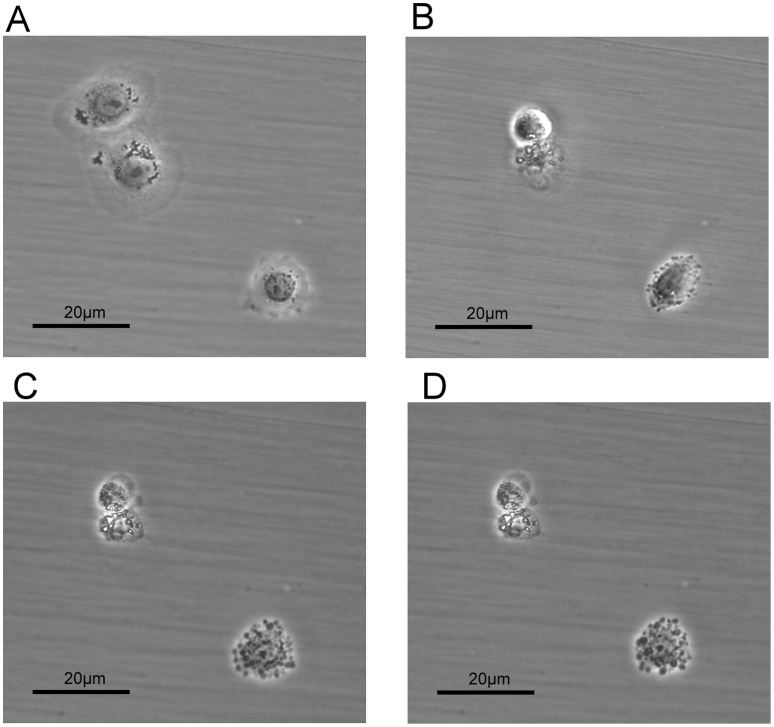
Liver cell morphology was affected by the administration of Gh-rTDH. The morphology of liver cells (FL83B) was clearly changed after the administration of 1 µg/ml Gh-rTDH for 24 hours at 37°C. The morphological changes included cell detachment and a loss of cell cytoplasm with cell shrinkage; they were the same cells that were recorded at different time points. Liver cells before (A) and after exposure to the Gh-rTDH protein for 8 hr (B), 16 hr (C), and 24 hr (D).

**Figure 3:**
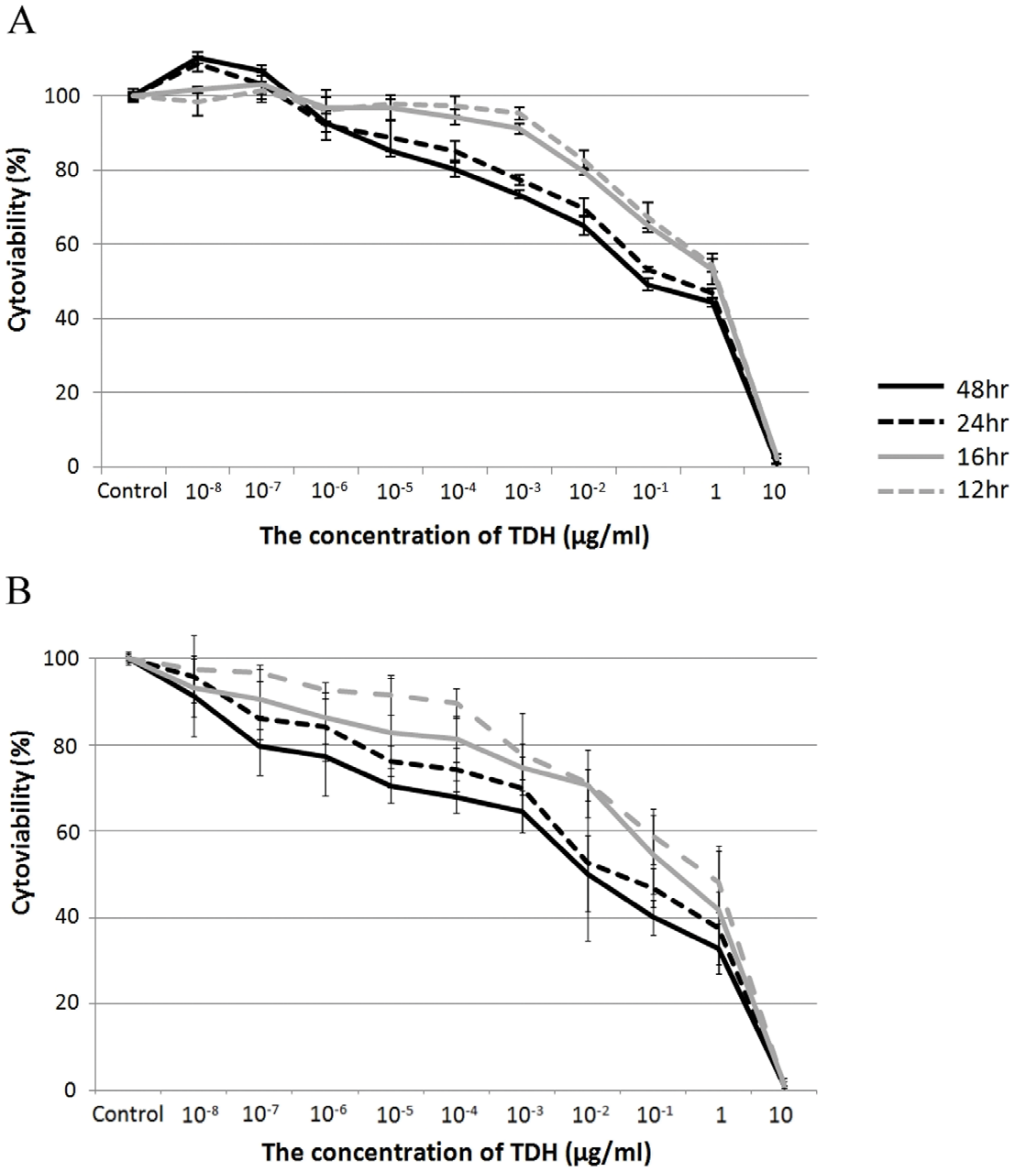
The MTT assay. The MTT assay revealed that the cytoviability of both (A) mouse and (B) human liver cells decreased in proportion to the concentration of Gh-rTDH over different treatment durations. Moreover, we noted that Gh-rTDH damaged liver cells *in vitro* when the concentration of Gh-rTDH exceeded 10^−6^ µg/ml.

Liver cell morphology was clearly affected by the administration of Gh-rTDH (Figure 2). In the control group (treated with PBS), we did not observe any morphological changes (or damage). The MTT assay revealed that the cytoviability of both mouse and human liver cells decreased in proportion to the concentrations of Gh-rTDH (Figures 3A and B). The hepatotoxicity caused by Gh-rTDH was both dose- and time-dependent. Very low concentrations of toxin (>10^−6^ µg/ml) caused damage to the liver cells.

#### 2.2 Gh-rTDH-FITC binds to the margins of liver cells and is taken up by their nuclei

**Figure 4:**
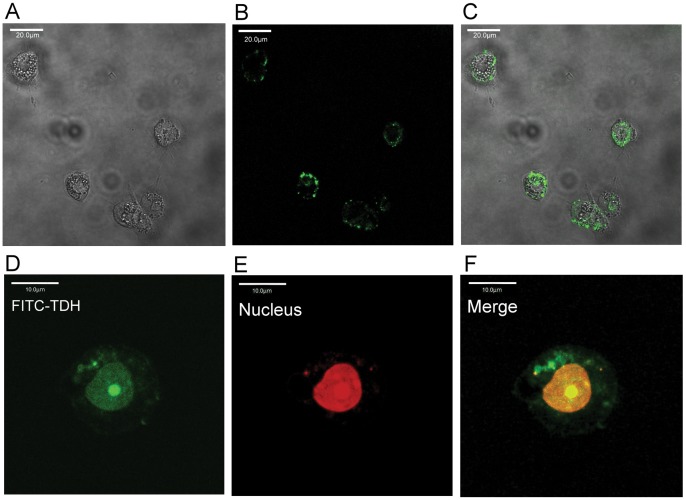
Subcellular localization of Gh-rTDH. Liver cells were treated with 10 µg/ml Gh-rTDH-FITC for 20 (A–C) or 40 (D–F) min at 26°C and were then observed by confocal microscopy. (A) The liver cells were observed without a FITC filter, (B) with a FITC filter, (C) and with A and B merged, confirming that Gh-rTDH-FITC (green) could bind around the liver cell margins. (D) Gh-rTDH-FITC (green) was taken up by the nuclei of liver cells. (E) Nuclei stained with PI (red). (F) The merge of D and E confirmed that Gh-rTDH-FITC was located in the nuclei of liver cells.

Gh-rTDH-FITC was bound around the margin of liver cells after the administration of 10 µg/ml Gh-rTDH-FITC for 20 min (Figures 4A–C). Moreover, Gh-rTDH-FITC was translocated to the nuclei of liver cells after treatment with Gh-rTDH-FITC for 40 min; the locations of the nuclei were confirmed by PI staining (Figures 4D–F).

### 3. Acute Hepatotoxicity was Noted by *in vivo* Analyses

#### 3.1 *In vivo* liver damage is quickly induced by Gh-rTDH

**Figure 5:**
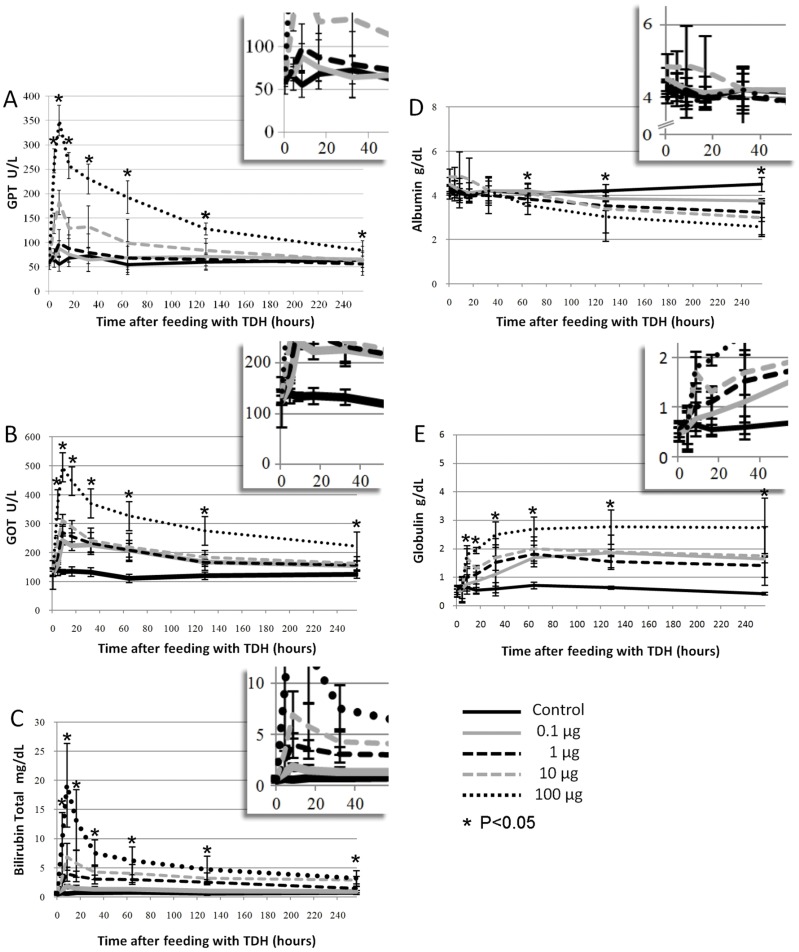
Liver function evaluation after a single administration of Gh-rTDH. The levels of liver function were abnormal after a single administration of Gh-rTDH. Six-week-old female BALB\c were treated with four different dosages of Gh-rTDH (0.1, 1, 10, and 100 µg), and the control group was treated with PBS (n = 5 for each group). Acute liver injury was demonstrated by elevated levels of (A) GPT and (B) GOT; the highest levels could be found at the 8^th^ hr after feeding in both. (C) Hyperbilirubinemia and (D) hypoalbuminemia also occurred in the mice that were treated with Gh-rTDH. The hyperbilirubinemia was the most severe at the 8^th^ hr, and hypoalbuminemia was noted after 32 hr of treatment with Gh-rTDH. (E) Globulin levels were gradually increased after exposure to Gh-rTDH. *A p-value <0.05 was considered statistically significant.

The levels of GOT and GPT were not elevated in the control group after the administration of a single dose of PBS. However, the mean GOT and GPT levels were clearly elevated in the group that was treated with 0.1 µg Gh-rTDH, and the highest levels were observed 8 hr after toxin administration. Similar findings were observed in other treatment groups. Higher doses of Gh-rTDH were clearly associated with more severe mouse liver injury (Figures 5A and B).

#### 3.2 Gh-rTDH induces an acute hemolytic status *in vivo*

**Figure 6:**
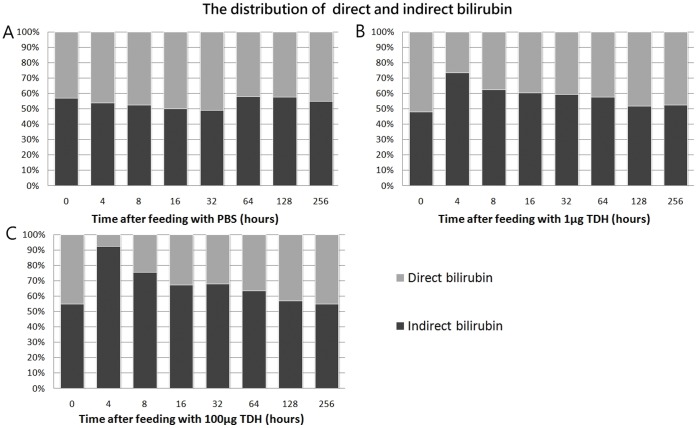
Gh-rTDH induces an acute hemolytic status. The distribution of direct and indirect bilirubin in mice that were fed with (A) PBS (control), (B) 1 µg of Gh-rTDH, or (C) 100 µg of Gh-rTDH.

Total bilirubin levels were clearly elevated in the groups that were treated with Gh-rTDH, and an increased dosage of Gh-rTDH resulted in further increases in total bilirubin levels (Figure 5C). Moreover, the proportions of indirect bilirubin were much higher than the direct bilirubin within 8 hr after exposure to 1 µg or 100 µg Gh-rTDH (Figure 6). Thus, an acute hemolytic status could be induced *in vivo* by the administration of Gh-rTDH.

#### 3.3 Gh-rTDH damages the albumin synthesis capacity of the liver and triggers the immune response

Albumin levels began to decrease at 32 hr after Gh-rTDH administration, and the decrease was proportional to the dosage. In the groups that were treated with Gh-rTDH, albumin levels progressively decreased and did not recover, even in the 256-hr treatment group (Figure 5D). These results indicate that albumin synthesis was damaged and did not recover during the initial 256 hr. By contrast, globulin levels were higher in the groups that received Gh-rTDH than in the control groups. This finding indicates that Gh-rTDH might trigger an immune system response in the circulation (Figure 5E).

#### 3.4 Gh-rTDH might not cause *in vitro* cardiotoxicity and nephrotoxicity

**Figure 7:**
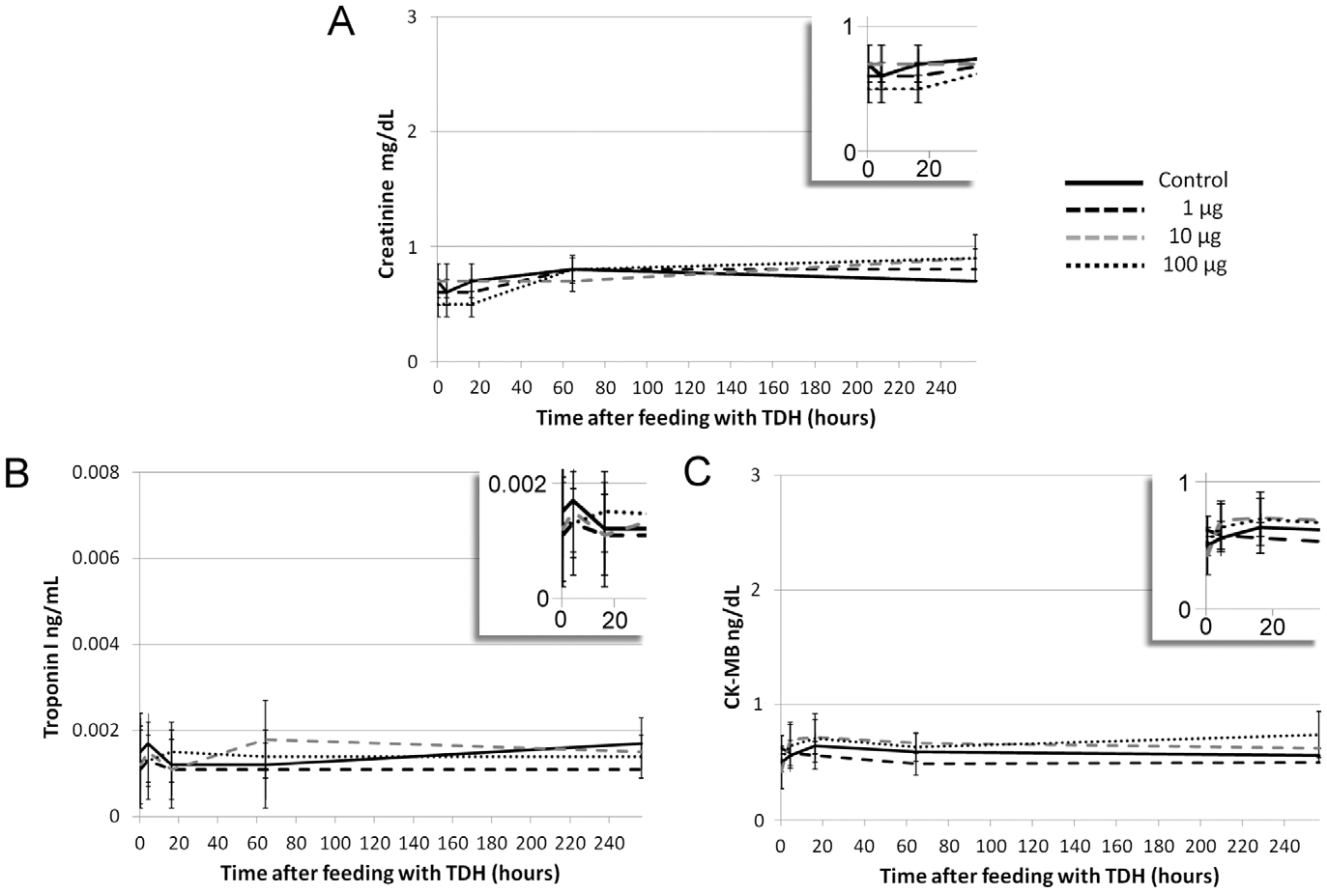
Gh-rTDH might not cause cardiotoxicity and nephrotoxicity. The levels of (A) creatinine, (B) CK-MB, and (C) troponin I did not significantly increase when mice were treated with different doses of Gh-rTDH.

Creatinine and CK-MB levels, which reflect kidney and heart injuries, were not elevated in Gh-rTDH-treated mice. The levels of creatinine and CK-MB did not change in proportion to the dosage of Gh-rTDH. The troponin I levels were also normal in all Gh-rTDH-treated mice (Figure 7).

#### 3.5 Hepatic damage is located in the periportal area of the liver

**Figure 8:**
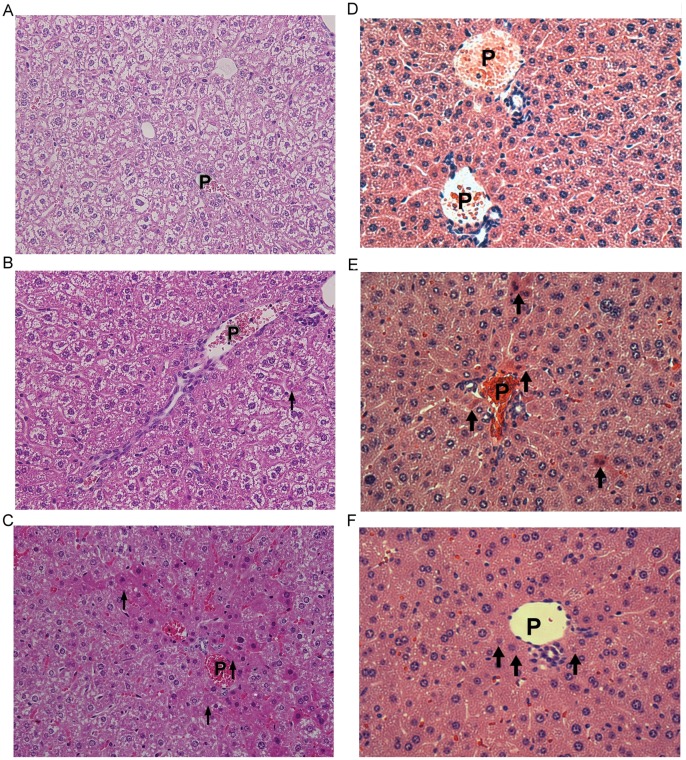
Liver biopsy (tissue harvested at the time of animal sacrifice) following Gh-rTDH ingestion and bacterial infection. Each image was acquired after 8 hr of toxin or bacterial exposure). The pathological images of liver parenchyma that were obtained from liver biopsies in mice were observed by microscopy at −400X (A, B, C) and −200X (D, E, F). (A) The parenchyma was homogeneous in mice treated with PBS (control group), and the liver cells around the portal vein were not damaged. (B) In mice treated with 10 µg Gh-rTDH, the parenchyma was mildly congestive over the periportal areas, and spotty damage (Councilman bodies) could be noted in the liver cells around the portal vein. (C) In mice fed 100 µg Gh-rTDH, the parenchyma was severely congestive with hemorrhage around the periportal areas, and most of the liver cells around the portal vein were damaged. These cells revealed confluent liver cell injury with intracytoplasmic acidophilic and ballooning change as well as nuclear pyknosis. Above all, these images demonstrated that Gh-rTDH was absorbed by the intestine and caused secondary injury to the liver via the venous return of the portal system. In mice fed *E. coli-T*OPO (D), the parenchyma was homogeneous and healthy. However, in mice fed *G. hollisae* (E) or *E. coli*-TOPO-*tdh* (F), cell damages were notable around the portal veins. P: portal vein, Arrows: damaged liver cells.

No pathological changes were noted in the liver parenchyma of the control group (Figure 8A). In mice treated with 10 µg Gh-rTDH, biopsies revealed the preservation of liver parenchymal architecture with mild congestion over the periportal areas and spotty liver cell damage around the portal vein. The damage was clearly located in the periportal area of the liver (zone 1 of the liver acinus) (Figure 8B). Moreover, severe congestion with hemorrhage was noted in mice that were treated with 100 µg Gh-rTDH (Figure 8C). Similar findings were noted for each mouse group that was biopsied.

#### 3.6^ 18^F-FDG PET/CT scans reveal decreases in and recovery of metabolism in the livers of treated animals

**Figure 9:**
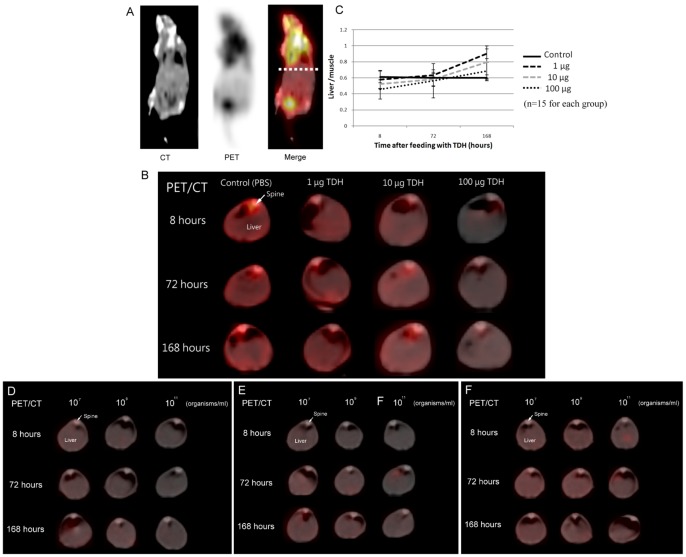
^18^F-FDG PET/CT scan. Mice were treated with 0.07 mCi ^18^F-FDG by tail vein injection, and imaging was performed 1 hr later. (A) All mice received 3 series of images, including CT, PET, and a merge of CT and PET. The location of the liver is labeled by a dotted line where the cross-sectioned images were obtained. (B) These images are cross-sections of the livers. At 8, 72 and 168 hr after treatment with Gh-rTDH, the uptake of ^18^F-FDG in livers decreased in proportion to the dosage of Gh-rTDH. (C) The ^18^F-FDG uptake value was calculated using the ROI (liver/muscle, semi-quantification) in each mouse. Higher doses of toxin indicated lower levels of ^18^F-FDG uptake. In the animal infection models, the ^18^F-FDG uptake levels were clearly lower in mice that were fed (D) *G. hollisae* or (E) *E. coli*-TOPO-*tdh* than those fed (F) *E. coli*-TOPO. These decreases were in proportion to the levels of bacteria in the treatment.

A series of 3 images was acquired for each mouse, including CT, PET, and a merge of the CT and PET after the ^18^F-FDG PET/CT scan. The red color in the merge images indicates ^18^F-FDG uptake by cells (Figure 9A). We found that hepatic ^18^F-FDG uptake in the livers of mice treated with Gh-rTDH was significantly lower than in mice that were given PBS; the decreased uptake was proportional to the dose of Gh-rTDH (Figure 9B). Moreover, we also noted that the ratios of liver/muscle ^18^F-FDG uptake levels clearly decreased at the 8^th^ hr after treatment with Gh-rTDH dose-dependently. In addition, the ratios of liver/muscle ^18^F-FDG uptake levels recovered to a normal range and even crossed the normal range during the 72^nd^ and 168^th^ hr after treatment with Gh-rTDH. These results indicate that liver glucose metabolism initially decreased after exposure to Gh-rTDH but that recovery continued for at least one week after a single exposure to the toxin (Figure 9C).

#### 3.7 *G. hollisae* and *E. coli*-TOPO-tdh but not *E.* coli-TOPO causes *in vivo* hepatotoxicity

GOT and GPT levels were not elevated after administration of *E. coli*-TOPO. However, the mean GOT and GPT levels were clearly elevated in the groups treated with *G. hollisae* or *E. coli-*TOPO*-tdh*, and the highest levels were observed 8 hr after bacterial treatment (data not shown). Higher concentrations of bacteria caused more severe liver injury. Acute hemolytic status, poor albumin synthesis, and more strongly induced immune system function were noted in mice that were fed *G. hollisae* and *E. coli*-TOPO-*tdh*. These patterns are similar to those observed in mice treated with Gh-rTDH. There was no pathological damage to the liver parenchyma in mice treated with *E. coli*-TOPO (Figure 8D). By contrast, spotty liver cell damage around the portal vein was noted in mice that were treated with *G. hollisae* (Figure 8E) or *E. coli*-TOPO-*tdh* (Figure 8F). The ^18^F-FDG PET/CT images revealed that the mice treated with *G. hollisae* (Figure 9D) or *E. coli*-TOPO-*tdh* (Figure 9E) exhibited much less ^18^F-FDG hepatic uptake than those treated with *E. coli*-TOPO (Figure 9F). Overall, the patterns of hepatotoxicity were notably similar between mice infected with *G. hollisae* or treated with Gh-rTDH. *E. coli*-TOPO did not cause significant liver injury.

## Discussion

In this study, both human and mouse liver cells were treated with *G. hollisae* TDH, and the *in vitro* hepatotoxicity was demonstrated by direct observation and the MTT assay. The hepatotoxicity caused by Gh-rTDH was both dose- and time-dependent. Very low concentrations of TDH (>10^−6^ µg/ml) damaged liver cells. We also noted that the MTT assays yielded a similar pattern over 12, 16, 24, and 48 hr under different toxin concentrations. One possible explanation is that when the concentration of toxin increased, cells were not only killed by this toxin but also probably suffered cell division suppression. Therefore, when we prolonged the treatment durations, the number of surviving cells did not clearly differ between the 4 time points. Naim et al. reported that *V. parahaemolyticus* TDH caused Rat-1 cell injury and that TDH might induce cytotoxicity by acting inside the cells [24]. In this study, we noted that the Gh-rTDH-FITC was taken up by liver cells via binding around the margin of the cell and was translocated to the nucleus within a short time period. Therefore, the destruction caused by *G. hollisae* TDH is notably quick and lethal to the liver cells.

Morros et al. firstly reported an account of a patient suffering a *G. hollisae* infection who presented with liver cirrhosis and hepatic encephalopathy in 1982 [25]. Previous studies demonstrated that *Vibrio* species infections could be more severe in patients with a history of impaired liver function [1], [9]. However, evidence of hepatotoxicity caused by *G. hollisae* TDH has never been provided. In this study, we found that the GOT and GPT levels in mice treated with Gh-rTDH obviously increased, and acute liver injury was strongly suspected. Muscle injury was not favored as the major reason for the elevation of GOT/GPT, as a ^18^F-FDG PET/CT scan did not show muscle injury. Moreover, liver biopsy revealed that the periportal areas of the livers were damaged and that the severity of the damage was associated with the dosage of Gh-rTDH. The periportal area in the liver is functionally well-known for its oxidative energy metabolism of fatty acids and amino acids, glucose release and glycogen formation, ammonia detoxification, protective metabolism, and the synthesis of albumin [26]–[28]. Therefore, mice with periportal area injury caused by Gh-rTDH might also suffer complications, which include malnutrition, protective system destruction, hepatic encephalopathy and hypoalbumenia. Clinically, the hypoalbumenia could be induced by decreased (hepatic) production or increased loss (gut tract loss). The result of hypoalbumenia in this study might be influenced by both mechanisms. The globulin levels also increased as the protective systems of the livers were damaged and the toxin triggered their immune systems in the circulation.

TDH is well-known for having strong hemolytic activity *in vitro* [9], [16], [24], [29]. We further noted that the acute hemolytic status *in vivo* arose within 4 hours after treatment with Gh-rTDH and increased in a dose-dependent manner. The acute hemolytic status *in vivo* results in acute anemia, which would exacerbate tissue hypoxia and organ hypoperfusion. Therefore, septicemia caused by *Vibrio* species with the *tdh* gene might be more critical than that caused by the *Vibrio* species without the *tdh* gene. Clinically, the hepatotoxicity might be caused via hemolysis. However, the pathological findings revealed that the hepatic injury was mainly located at the periportal areas, and the injury was not diffused. It is suspected that the major etiology is toxin absorption and injury to the liver via the venous return of the portal system.

Clinical ^18^F-FDG PET/CT scans have been reported as excellent tools to survey organ metabolism in small animals [30]. Damage in the liver caused by Gh-rTDH can be demonstrated by blood withdrawal and liver biopsy. However, the conditions of recovery and organ metabolism in living animals were difficult to analyze. Therefore, ^18^F-FDG PET/CT scans were performed for our assessment. We noted that the uptake of ^18^F-FDG in the livers decreased in proportion to the administered dosages of Gh-rTDH, which indicate that the hepatic damage in the animals was dose-dependent. In other non-hepatic organs, damage was not obvious.

After exposure to Gh-rTDH, the uptake of ^18^F-FDG gradually increased in trend. We suggest that the livers could finally reconstruct from the destruction of Gh-rTDH exposure, and these liver cells had undergone repair and proliferation via increasing their uptake of glucose, which is well-known as an unavoidable material in metabolism. The metabolism of glucose in the livers damaged by Gh-rTDH almost recovered to a normal range in the 72^nd^ hour after exposure to TDH. Furthermore, the metabolism of glucose crossed the normal range in the 168^th^ hour after exposure to Gh-rTDH, and the recovery was more predominant in mice treated with low dosages than in those treated with a high dosage of Gh-rTDH. The level of glucose uptake crossing the normal range noted that the metabolism of glucose was notably robust in these damaged livers in addition to ongoing strong recovery. According to our findings from the liver biopsies, the construction might be mainly located in the periportal area, which has been labeled as a major location of glucose and amino acid metabolism [26]–[28]. Therefore, the construction in the periportal area might contribute to the high level of ^18^F-FDG intake in the liver during the recovery stage. Overall, this finding might provide strong evidence indicating that the reconstruction of liver continues for at least one week after a single Gh-rTDH exposure and that the damaged liver has the ability to recover from the Gh-rTDH related injury, even when exposed to a massive dosage of Gh-rTDH. Consistent with this observation is the finding that differential hepatotoxicity could be detected when mice were treated with different amounts of *G. hollisae and E. coli*-TOPO-*tdh* but were free from hepatotoxicity with *E. coli*-TOPO. The ^18^F-FDG PET/CT results of the animal infection models showed that the severity of the liver injury was notably similar in mice treated with 100 µg of Gh-TDH and in mice treated with 10^10^ organisms of *G. hollisae*. Therefore, we suspected that 10^8^ organisms of *G. hollisae* might produce 1 µg of TDH and cause liver injury *in vivo*. The results clearly demonstrate the *in vivo* hepatotoxicity of the *Gh-tdh* gene product.

In conclusion, *G. hollisae* TDH is reported as having *in vitro* and *in vivo* hepatotoxicity in our study. *G. hollisae* TDH damaged the liver in living animals and mainly attacked the periportal area, which is associated with the synthesis of albumin and the metabolism of glucose. Most importantly, the ^18^F-FDG PET/CT scan revealed evidence that the reconstruction of the liver continued at least for one week after a single exposure of *G. hollisae* TDH. Furthermore, the damaged liver was shown to have an adequate ability to recover.
